# Liquid Biopsy in Whole Blood for Identification of Gene Expression Patterns (mRNA and miRNA) Associated with Recurrence of Glioblastoma WHO CNS Grade 4

**DOI:** 10.3390/cancers16132345

**Published:** 2024-06-26

**Authors:** Razan Muhtadi, Denise Bernhardt, Gabriele Multhoff, Lisa Hönikl, Stephanie E. Combs, Sandro M. Krieg, Jens Gempt, Bernhard Meyer, Vahé Barsegian, Monika Lindemann, Mandy Kasper, Samantha Stewart, Matthias Port, Michael Abend, Christian D. Diehl, Patrick Ostheim

**Affiliations:** 1 Bundeswehr Institute of Radiobiology, 80937 Munich, Germany; razan_muhtadi@gmx.de (R.M.); mandykasper@bundeswehr.org (M.K.); samantha1stewart@bundeswehr.org (S.S.); matthiasport@bundeswehr.org (M.P.); michaelabend@bundeswehr.org (M.A.); 2 Graduate Center of Medicine and Health, Technical University Munich, 81675 Munich, Germany; 3 Department of Radiation Oncology, TUM School of Medicine and Health, Technical University of Munich (TUM), 81675 Munich, Germany; denise.bernhardt@mri.tum.de (D.B.); gabriele.multhoff@tum.de (G.M.); stephanie.combs@tum.de (S.E.C.); 4 Department of Neurosurgery, School of Medicine, Klinikum Rechts der Isar, Technical University of Munich (TUM), 81675 Munich, Germany; lisa.hoenikl@mri.tum.de (L.H.); sandro.krieg@tum.de (S.M.K.); jens.gempt@tum.de (J.G.); bernhard.meyer@tum.de (B.M.); 5 Deutsches Konsortium für Translational Krebsforschung (DKTK), Partner Site Munich, 80336 Munich, Germany; 6 Institute of Radiation Medicine (IRM), Helmholtz Zentrum München GmbH, German Research Centre, 85764 Oberschleißheim, Germany; 7 Department of Neurosurgery, University Hospital Heidelberg, Heidelberg University, 69117 Heidelberg, Germany; 8 Department of Neurosurgery, University Medical Center Hamburg-Eppendorf, 20251 Hamburg, Germany; 9 Institute of Nuclear Medicine, Helios Kliniken, 19055 Schwerin, Germany; vahe.barsegian@helios-gesundheit.de; 10 Institute for Transfusion Medicine, University Hospital Essen, 45147 Essen, Germany; monika.lindemann@uk-essen.de

**Keywords:** liquid biopsy, glioblastoma WHO CNS Grade 4, gene expression, mRNA, miRNA, whole-genome screening

## Abstract

**Simple Summary:**

This research investigates the identification of tumor-specific serological biomarkers for the early diagnosis of glioblastoma multiforme (GBM) recurrence using peripheral whole blood samples in a minimally invasive approach known as “liquid biopsy”. Our results indicate that gene expression analysis can detect changes in transcriptional (mRNAs) and post-transcriptional (small RNAs) levels after surgery and upon tumor recurrence. This prospective study aims to develop a diagnostic tool complementary to MRI modalities in the clinical follow-up, helping to track tumor progression/recurrence and to make clinical decisions.

**Abstract:**

GBM WHO CNS Grade 4 represents a major challenge for oncology due to its aggressive behavior. Conventional imaging has restrictions in detecting tumor recurrence. This prospective study aims to identify gene-based biomarkers in whole blood instead of isolating exosomes for the early detection of tumor recurrence. Blood samples (n = 33) were collected from seven GBM patients at time points before and after surgery as well as upon tumor recurrence. Four tumor tissue samples were assessed in parallel. Next-generation sequencing (NGS), including mRNA-seq and small RNA-seq, was used to analyze gene expression profiles in blood samples and tumor tissues. A novel filtering pipeline was invented to narrow down potential candidate genes. In total, between 6–93 mRNA and 1–19 small RNA candidates could be identified among the seven patients. The overlap of genes between the patients was minimal, indicating significant inter-individual variance among GBM patients. In summary, this prospective study supports the applicability of gene expression measurements in whole blood for the detection of tumor recurrence. It might provide an alternative to the challenging workflow of liquid biopsy after laborious exosome isolation from whole blood.

## 1. Introduction

Glioblastoma multiforme (GBM) is one of the most aggressive types of cancer, with a poor prognosis, despite advanced treatment regimens and intensive research [1,2]. In adults, malignant gliomas account for about 70% of primary brain tumors. The typical survival duration ranges from 12 to 15 months, and less than 3 to 7% of patients survive longer than five years [1,3]. Standard treatment following the “Stupp Scheme” involves surgical resection of the tumor to a safely feasible extent, followed by adjuvant temozolomide-based (TMZ) radiochemotherapy (RCTx) [4]. However, due to the progressive nature of this malignancy, the prognosis remains dismal, with a very high rate of local recurrence. The aggressiveness, drug resistance, and recurrence are GBM characteristics. Reasons for that can be the high cell proliferation rate, genetic instability, increased angiogenesis, and the fact that residual tumor cells infiltrate into the healthy brain parenchyma, the main tumor mass [5]. Its boundaries can hardly be detected morphologically, making complete surgical resection challenging [6].

In addition to novel diagnostic modalities like advanced Magnetic Resonance Imaging (MRI) modalities and 18-fluoride-fluoro-ethyl-tyrosine positron emission tomography (FET-PET) imaging, common structural MRI remains the cornerstone of imaging evaluation in the whole clinical history of GBM patients [7]. It plays a key role in initial diagnosis, post-surgery follow-ups, and especially in the diagnosis of tumor recurrence. However, it is of limited value when having to differentiate treatment-related changes from surgery, chemo- and radiotherapy, also known as pseudoprogression, and tumor recurrence [8]. The therapy-associated appearance of tissue necrosis, granulation tissue, and post-operative scar on conventional contrast agent-weighted MRI images may be similar to the residual tumor or a tumor recurrence [9].

Due to these considerations, tumor biopsy as the “gold standard” remains the most predictable approach for differentiating treatment-associated changes from tumor recurrence when imaging is unclear. However, taking a tumor biopsy is highly invasive, with all surgery-associated risks for the patient, like brain swelling, hemorrhages, infarction, etc., affecting the patient’s quality of life [9]. The ability to accurately detect tumor progression in a more sensitive manner is needed for GBM patients. Hereby, tumor-specific serological biomarkers represent an exciting area of research that holds promising tools for GBM therapy. Circulating biomarkers have the advantage of being tumor-specific and providing a “fingerprint” at the gene expression level in the blood that will help with the early diagnosis of GBM [10,11]. Extracellular vesicles (EVs) such as exosomes and microvesicles have proven to be reliable markers in certain tumors like pancreatic and renal cell carcinoma [12,13,14] but also in GBM [15,16,17]. Those EVs ranging in size from 40 to120 nm contain proteins, lipids, and various types of RNA (exosomal mRNA, miRNA, lncRNA). They play a crucial role in cell–cell communication, e.g., regulating tumor growth, metastasis, and angiogenesis in cancer development [18]. EVs, with their distinct cellular cargo, provide a comprehensive view of the cell’s status at the time of release [19]. This unique feature has paved the way for the concept of using EVs for a ‘liquid biopsy’ from tumor cells, offering a potential breakthrough in cancer diagnosis and disease monitoring [15]. This offers the possibility to isolate and analyze tumor material non-invasively. Such a method provides the possibility to enable the early monitoring and detection of cancer recurrence, helping clinicians to make early therapy decisions [20]. Nevertheless, isolation of EVs from peripheral blood is challenging (e.g., long-lasting ultracentrifugation and low amounts of isolated RNA [21,22]) and difficult to establish in the daily clinical routine [21]. A more practical but less examined approach is using whole blood (PAXgene Blood RNA tubes, Qiagen, PreAnalytiX GmbH, Hilden, Germany) for tumor-specific serological biomarkers.

**Figure 1:**
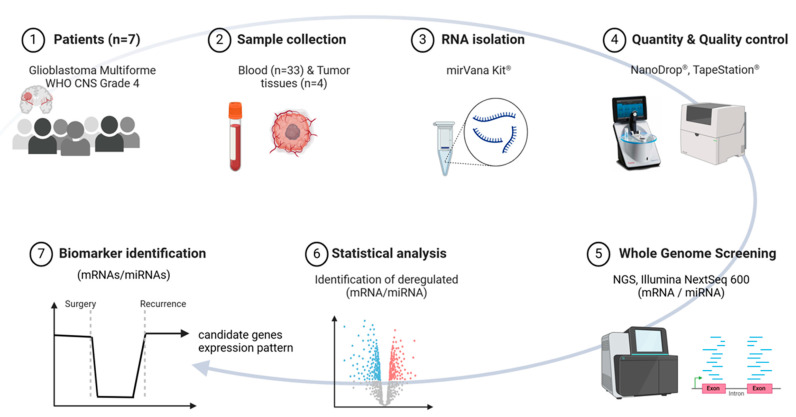
Shows the study design for this pilot study, starting with (1) the enrollment of seven patients diagnosed with Glioblastoma WHO CNS Grade 4, (2) the number of tumor tissues and blood samples collected, (3) RNA isolation for tumor tissues and blood samples via mirVana^®^ kit, (4) quantity and quality controls for the isolated RNA, (5) whole-genome screening for mRNAs and miRNAs using next-generation sequencing techniques, and finally (6,7) identification of deregulated mRNAs and miRNAs (biomarkers) by a triple-filtering method applied over the whole clinical follow-up until the confirmation of tumor recurrence via MRI. Abbreviation: NGS = next-generation sequencing; MRI = Magnetic Resonance Imaging. Created with https://Biorender.com accessed on 14 May 2024.

This study aims to explore the minimally invasive early diagnosis of tumor recurrence by detecting tumor-specific serological gene signatures in the whole blood of patients with a GBM WHO CNS Grade 4. Blood samples were collected before and at specific time points after initial tumor resection during the clinical course, and tumor tissue was preserved. Gene expression analysis at a transcriptional (protein-coding mRNAs) and a post-transcriptional (non-coding small RNAs) level was conducted for each patient using whole-genome screening through next-generation sequencing (NGS). Comparing pre-surgery and post-surgery blood samples is expected to reveal a decrease in potential candidate mRNA and miRNA species associated with reduced or absent tumor cells. In GBM recurrence, an increase in these identified tumor markers in peripheral blood is anticipated (Figure 1). The purpose is to provide a complementary diagnostic tool to the serial MRI modalities as a novel way to track tumor progression, pseudoprogression, and treatment monitoring, thus helping radiologists and clinicians to be more confident in clinical decision making.

## 2. Materials and Methods

### 2.1. Patients, Treatment, Ethical Approvals, Guidelines

Seven patients with histologically proven glioblastoma WHO CNS Grade 4 (World Health Organization classification) in accordance with the revised 2021 criteria were included in this prospective study. The WHO criteria were changed during the study, and we ensured that all patient diagnoses met the 2021 WHO criteria. All patients were treated at the Klinikum rechts der Isar, Technical University of Munich (TUM), Munich, Germany, during a four-year time period (2019–2023). Inclusion criteria involved patients aged ≥18 years who were not taking immunosuppressive substances. Patients with long-term therapy of high-dose COX inhibitors, histone acetylation or DNA methylation-affecting substances (e.g., valproic acid), and tricyclic antidepressants were excluded. In the current pilot study, only patients with documented tumor recurrence after therapy and during the follow-up period were considered.

All patients underwent similar standard therapy following the “Stupp Scheme”, which involves surgically removing the primary tumor to the safely feasible extent, followed by radiotherapy (intensity-modulated radiation therapy, IMRT) for a total of 60 Gy given in daily fractions of 2 Gy for five days per week over six weeks in total, plus continuous daily temozolomide (TMZ) 75 mg/m^2^/kg for seven days per week. After radiotherapy, a further six adjuvant TMZ cycles (150 to 200 mg/m^2^/kg for five days each month) were given. Additionally, three patients received intraoperative radiotherapy (IORT). In this prospective study, all patients developed local tumor recurrence 158.3 (SD ± 79.3) days after the initial surgery.

Ethical approval for the study was secured from the responsible Ethics committee of the Technical University, Munich, Germany (approval number 572/16 S), before the start of the study. Written informed consent was obtained from each participating patient. All methods described in this manuscript (e.g., RNA quantity/quality and TaqMan qRT-PCR) were performed according to the relevant guidelines, regulations, and standard operating procedures implemented at the Bundeswehr Institute of Radiobiology in 2008 when the Institute became DIN-accredited by TÜV Süd München, Germany (DIN EN ISO 9001/2008), and were described in detail elsewhere [23,24]. All data were managed in compliance with the European General Data Protection Regulation.

### 2.2. Sample Collection: Whole Blood and Tissue

Whole blood samples (2.5 mL) were obtained through venipuncture and collected in PAXgene Blood RNA tubes (BD Diagnostics PreAnalytiX GmbH, Hombrechtikon, Switzerland), ideally at the following time points (Table 1): (1) 1–5 days before surgery, (2) 5–9 days after surgery, (3) on the first day of RCTx (2nd–4th week after surgery/19–29 days after surgery), (4) four weeks after the end of the RCTx (84–97 days after surgery), (5) in three-month intervals following the completion of RCTx, and (6) directly before or after diagnosis of tumor recurrence (−13–17 days after diagnosis of tumor recurrence). The tubes were gently inverted (10 times), kept at room temperature overnight, and then stored at −20 °C.

Tumor tissue biopsies of the GBM tumors were taken during primary surgery, incubated immediately in RNAlater solution (Qiagen, Hilden, Germany), and stored at −20 °C. The PAXgene tubes and tissue samples were transported on ice to the Bundeswehr Institute of Radiobiology for further processing.

### 2.3. RNA Extraction and Quality Control

PAXgene Blood RNA system was used to extract RNA from blood samples. After thawing the PAXgene tubes followed by washing and centrifugation steps, cells in the supernatant were lysed (Proteinase K), and a Lysis/Binding Solution taken from the mirVana kit (Life Technologies, Darmstadt, Germany) was added. With the mirVana kit (Life Technologies, Darmstadt, Germany), the total RNA, including small RNA species, was isolated by combining a phenol-chloroform RNA precipitation with further processing using a silica membrane. After several washing procedures, DNA residuals became digested on the membrane (RNAse free DNAse Set, Qiagen, Hilden, Germany). RNA was eluted in a collection tube and frozen at −20 °C.

RNA isolation from 10–20 mg tumor tissue was conducted by mixing the tissues with RLT—ß-mercaptoethanol and 5 mm stainless-steel beads (Qiagen, Hilden, Germany). The mixture was disrupted using TissueLyser LT (Qiagen, Hilden, Germany) at 50 Hz for 3 min. Subsequently, cells in the supernatant were lysed (Proteinase K), followed by the addition of Lysis/Binding Solution taken from the mirVana kit (Life Technologies, Darmstadt, Germany), according to the previously mentioned steps.

The quality and quantity of isolated total RNA were measured spectrophotometrically (NanoDrop, PeqLab Biotechnology, Erlangen, Germany). RNA integrity was assessed using both the 4200 TapeStation System and the 2100 Agilent Bioanalyzer (both Agilent Technologies, Santa Clara, CA, USA). DNA contamination was checked by performing PCR with an actin primer pair. Only RNA samples with an A260 nm/A280 nm ratio ≥ 2.0 (Nanodrop) and RNA integrity number (RIN) ≥ 7 for blood samples and (RIN) ≥ 6 for tumor tissues were utilized for NGS library preparation (IMGM Laboratories, Martinsried, Germany).

### 2.4. Whole-Genome Screening (mRNAs and miRNAs)

Whole-genome screening (next-generation sequencing, NGS) at a transcriptional (mRNA seq) and a post-transcriptional (small RNA seq) level was performed for the detection of differentially expressed genes. From the 7 patients, 33 blood samples (pre-surgery, post-surgery, and post-recurrence) and 4 tumor tissue samples were used (Table 1). Three tumor tissue samples from the remaining patients were excluded from this study due to the complete degradation of RNA.

#### 2.4.1. Library Preparation

mRNA sequencing libraries were prepared utilizing the TruSeq^®^ Stranded Total RNA Kit, with Ribo-Zero globin depletion (Illumina, San Diego, CA). This process involved depleting cytoplasmic and mitochondrial rRNA as well as globin mRNA following the manufacturer’s recommendations (Illumina Inc. TruSeq^®^ Stranded Total RNA Sample Preparation Guide—Catalog #RS-122-9007DOC—Part #15031048 Rev. E. 2013). The depleted RNA was fragmented, followed by cDNA synthesis, 3′ends adenylating, and ligation of sequencing adapters containing primer and flow cell binding sites. Subsequently, library quantification using Qubit^®^dsDNA HS Assay Kit (Thermo Scientific, Waltham, MA, USA) and assessment of size distribution by using Agilent 2100 Bioanalyzer (Agilent, Santa Clara, CA, USA) were performed. Finally, the library pool comprising single-stranded DNA fragments with sequencing adapters, sequencing primer binding sites, and indices underwent sequencing.

For small RNA sequencing libraries, the NEBNext^®^ Small RNA Library Prep Kit for Illumina (Illumina, San Diego, CA, USA) was employed following the manufacturer’s recommendations (New England Biolabs Inc., Ipswich, MA, USA, NEBNext^®^ Small RNA Library Prep Set for Illumina (Multiplex Compatible)—Catalog #E7330S/L—Version 4, 9/17). Briefly, RNA was fragmented, followed by reverse transcription to synthesize cDNA. Fragments were adenylated and ligated with sequencing adapters containing primer and flow cell binding sites, as well as indices for multiplex sequencing. Subsequently, library quantification and size distribution assessment were performed using the Qubit^®^dsDNA HS Assay Kit (Thermo Scientific, Waltham, MA, USA) and the Agilent 2100 Bioanalyzer (Agilent, Santa Clara, CA, USA), respectively. Before sequencing, adapter dimers were removed by gel electrophoresis using 3% low-range agarose (BioRad, Hercules, CA, USA) gel stained with sensitive SYBR Green dye (Thermo Fisher Scientific). The gel fragments were purified using the MinElute Gel Extraction Kit (Qiagen), followed by additional quality control using library quantification and electrophoresis, as described above.

#### 2.4.2. Sequencing Platform and Data Preprocessing

Prepared libraries for mRNAs and miRNAs were sequenced on the Illumina NovaSeq^®^ 6000 sequencing system (Illumina, San Diego, CA, USA), with exclusion amplification (ExAmp) chemistry, utilizing the sequencing by synthesis approach with two-channel signal detection. After importing read data into the analysis tool, sequence reads were mapped against the human reference genome GRCh38/hg38 (NCBI) and mirRBase Release 22. For in-depth analysis of differential gene expression and annotation of reads, the STAR aligner was utilized for mRNAs, and CLC Genomics Workbench (CLC Bio-Qiagen, Aarhus, Denmark; version 21.0.5) was employed for miRNAs.

#### 2.4.3. Filter Criteria for Identification of Potential Candidate Genes

**Figure 2:**
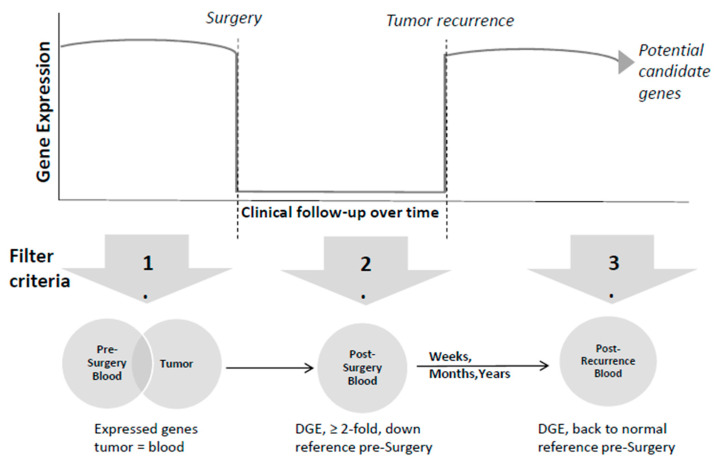
Represents the filtering methods employed to identify the potential candidate genes. The upper part shows the gene expression during clinical follow-up, including pre-surgery, post-surgery, and tumor recurrence phases. The lower part shows the start of the gene selection process comprising the total number of detected mRNAs and miRNAs expressed in both tumor and blood before surgery. Subsequently, mRNAs and miRNAs exhibiting at least 2-fold downregulation post-surgery compared to pre-surgery levels (fold change, FC ≤ 0.5) were selected. Following tumor recurrence, which can occur over different periods, these candidate genes are expected to return to pre-surgery levels or even exceed them (FC ≥ 1). Abbreviation: DGE = differential gene expression.

To identify potential candidate mRNA and miRNA species, we sequentially applied specific filter criteria to the expression data of all patients. This filtering process comprised three stages (Figure 2):

1. mRNAs/miRNAs had to be expressed (≥10 normalized counts using DESeq2’s median of ratios) in both tumor and blood samples prior to surgery (pre-surgery). In case the tumor material was of insufficient quality and had to be excluded from further analysis, the first filter implied mRNAs/miRNAs expressed (≥10 normalized counts) in pre-surgery blood only.
2. When comparing pre-surgery with post-surgery blood samples, a decrease in the copy numbers of potential candidate mRNAs and miRNAs was expected. A second filter searching for at least 2-fold downregulated mRNAs/miRNAs in >50% of samples after surgery compared to pre-surgery associated with reduced or absent tumor cells (post-surgery) was introduced.
3. In case of tumor recurrence, a third filter enabled the search for upregulated mRNAs/miRNAs, resulting in gene expression (GE) levels comparable to pre-surgical samples or even higher expression levels (post-recurrence). This assumed an increase in these potential tumor markers in the peripheral blood.

Only genes passing all three filters over the whole clinical follow-up were considered as potential candidate mRNAs/miRNAs for being associated with GBM recurrence.

### 2.5. Bioinformatics

Primary-image processing was conducted using Real Time Analysis 3.4.4 Software (RTA) and bcl2fastq 2.20.0.422. The Illumina Sequence Analysis Viewer (SAV) 2.4.7 assessed sequencing run performance, while NovaSeq^®^ 6000’s inherent NCS and RTA software handled image and signal processing. CLC Genomics Workbench v.21.0.5 facilitated small RNA analysis, covering extraction, counting, annotation, and merging using miRBase Release 22. DESeq2 in R software V. 4.1.2 was used to normalize counts using the median of ratios method and determine fold changes for small RNA classification. Only small RNAs with a minimum of 10 normalized counts were considered for further analysis. A small RNA is classified as regulated if its FC value exceeds 2 or is less than 0.5.

The analysis of mRNA involved exon-based expression analysis. The STAR aligner mapped mRNA-Seq data to the hg38 reference sequence, and HOMER’s annotatePeaks.pl tool counted exons, with subsequent bioinformatics filtering in R software ensuring the inclusion of exons, with a minimum of 10 counts per sample. An average of 4.2 × 10^6^ exon reads per sample could be achieved.

For potential candidate genes, we performed gene set enrichment analysis using PANTHER pathway software [http://www.pantherdb.org/, accessed on 11 June 2024, version 18.0, Released 17 September 2023]. PANTHER categorizes genes by similar biological functions based on their annotation [reference list was the current homo sapiens gene ontology (GO) database]. The analyses included PANTHER GO-Slim Cellular Component, PANTHER Protein Class, PANTHER Pathways, and GO Molecular Function Complete. The False-Discovery Rate (FDR) algorithm corrected *p* values for multiple testing (Table 3). Details on gene names, symbols, persistent IDs, orthologs, Panther family/subfamily or Panther protein class, and their association with GBM are provided in Supplemental Table S2.

### 2.6. Statistical Analysis

Excel was used for descriptive analysis and for generating the tables. Samples of each patient were examined separately. The graphical illustrations were created utilizing SPW (SigmaPlot, Version 14.5, Jandel Scientific, Erkrath, Germany) and PowerPoint (Microsoft, Version 16.0.4266.1001, Redmond, DC, USA).

## 3. Results

### 3.1. RNA Quantity and Quality

In total, 33 blood samples and 4 tumor tissues from seven GBM WHO CNS Grade 4 patients (five males, two females, mean age 57.9 ± 13.6 years) were provided for analysis (Table 1). In pre-surgery blood samples, 8.7 µg (SD ± 3.6) total RNA was isolated, with a mean RNA integrity number (RIN) of 7.8 (SD ± 0.7). In all blood samples after surgery until the stage of recurrence, an average of 6.5 µg (± 5.6) total RNA was isolated with an average RIN of 8.1 (SD ± 0.5). From blood samples after diagnosis of tumor recurrence, an average of 5.4 µg (SD ± 2.8) total RNA was isolated, with an average RIN of 7.7 (SD ± 0.7), indicating sufficient amounts of high-quality RNA for NGS analysis. The β-actin PCR could not detect DNA contamination in any sample 

On average, 37.1 µg (SD ± 22.8) of total RNA was isolated from tumor tissues. The RIN for tumor tissue RNA samples showed a mean value of 7.8 (SD ± 1.2) using the 2100 Agilent Bioanalyzer. Three tumor tissue samples were excluded from further analysis because of poor quality or even degradation 

#### Library Quantity and Quality

Electropherograms confirmed the absence of rRNA in depleted RNA samples and exhibited typical fragment distributions for mRNA libraries, indicating successful amplification. Quantification using the Qubit^®^ ds DNA HS Assay kit confirmed sufficient DNA amounts for sequencing across all libraries, on average 26.6 ng/µL for total RNA and 23.9 ng/µL for small RNA. The mean Q30 bases for the runs were 92.8%, 93.5%, and 91.0%. The average number of passed filter (PF) reads from total RNA was 23.2 × 10^6^ and 25.6 × 10^6^ for miRNA.

### 3.2. Gene Expression Analysis—Detection of Potential Candidates

Combining all three filters ((1) including expression in pre-surgery blood/tissue samples, (2) downregulation in post-surgery samples, and (3) upregulation to pre-surgical levels post-recurrence; Figure 2) narrowed down all identified genes to potential candidate genes. In total, between 6–93 potential candidate genes could be identified at the transcriptional level (mRNAs) among the seven patients and 1–19 at the post-transcriptional level (small RNAs) (Table 2). The decrease in genes after surgery was emphasized at later time points after surgery. The majority of the candidates exhibited a 2-5-fold decrease in RNA copy numbers post-surgery (Supplemental Table S1).

The filtering process to further refine the selection of suitable candidate genes is exemplarily described for patient #1, as shown in Table 2; from 1.4 × 10^5^ normalized exons and 622 small RNAs after applying the first filter criterion (expression in pre-surgery blood and tumor tissue), 567 mRNAs, along with 186 small RNAs could be identified. Subsequently, 188 mRNAs, along with 58 small RNAs, were differentially downregulated (>2-fold in >50% of samples after surgery compared to pre-surgery) associated with reduced or absent tumor cells at post-surgery time points (second filter criterion ). At the time of diagnosis of tumor recurrence, 93 mRNAs and 19 small RNAs were upregulated again to pre-surgery levels or even increased expression (third filter criterion). Those are thought to be potential candidate genes (Table 2).

**Figure 3:**
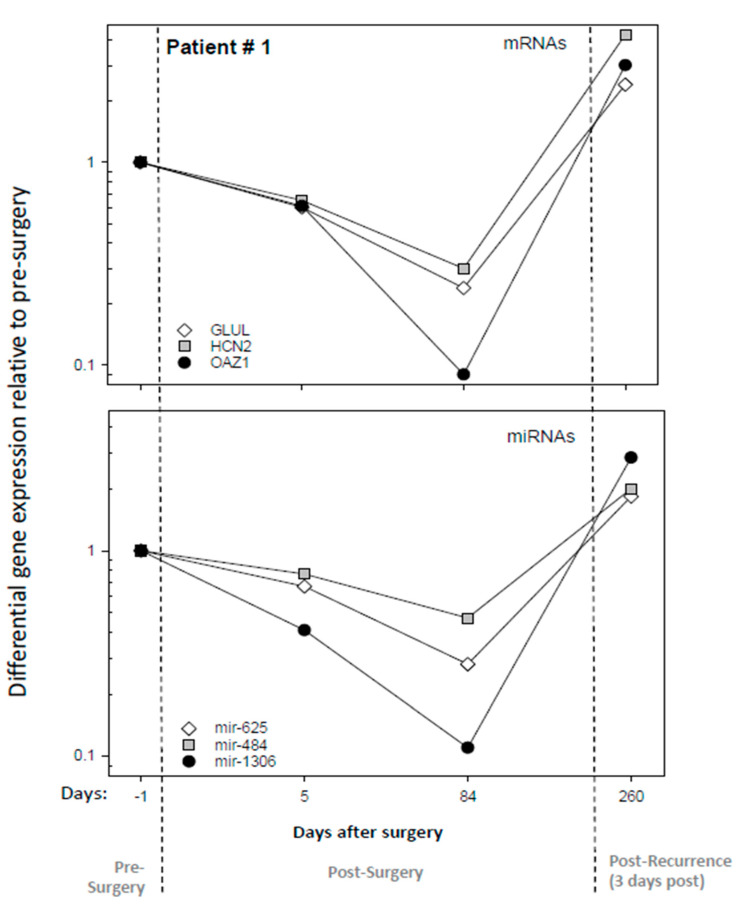
Illustrates the differential gene expression (DGE) results for representative mRNAs and miRNAs species during the pre-surgery, post-surgery, and tumor recurrence phases for patient number one. The upper panel shows DGE relative to the pre-surgery sample of three promising mRNAs (*GLUL, HCN2*, and OAZ1) 1 day before surgery, 5 and 84 after surgery, and 3 days after recurrence (260 days post-surgery). The lower panel shows the DGE of three promising miRNAs (*mir-625, mir-484*, and *mir-1306*) across the same time points. Gene names are listed in all graphs.

Representative examples (three mRNAs and three miRNAs from patient #1) for the time-dependent downregulation after surgery and an upregulation to pre-surgery levels or even increased expression at the time of tumor recurrence are shown in Figure 3. Five days following surgery, the mRNA (*OAZ1*) and the miRNA (*mir-1306*) expression levels showed about a 2-fold decrease in fold change, with values of 0.6 and 0.4, respectively. A further almost 10-fold decrease (0.09- and 0.1-fold changes) was observed after completion of RCTx on day 84 post-surgery. Three days after tumor recurrence, fold changes of 3 and 2.9 above pre-surgery were examined, respectively.

In Patient #5, we noticed that specific candidate genes, such as *SORL1*, *SELL*, and *KMT2D*, showed an almost 10-fold decrease on days 97 and 184 post-surgery. Remarkably, 13 days prior to tumor recurrence, which was later confirmed by MRI scans, the same genes were subsequently upregulated to levels that matched or exceeded those of the pre-surgery sample (Supplemental Table S1).

**Figure 4:**
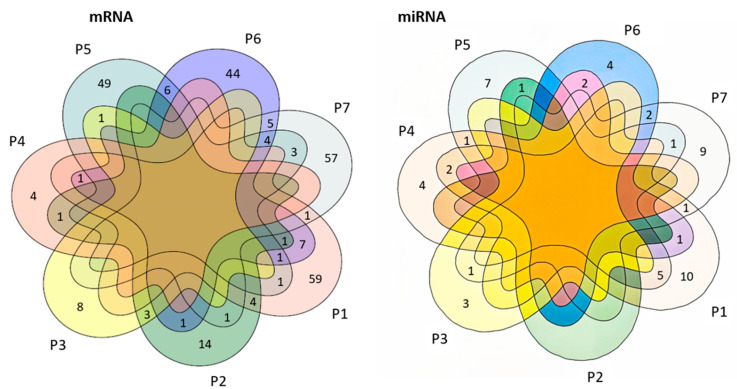
Venn diagrams illustrate the number of detected candidate mRNAs (**left part**) and miRNAs (**right part**) for the seven patients and their overlap. Numbers outside the overlapping region represent RNAs that were not in common for other time points. Overlapping circles represent the number of RNAs that were in common with multiple patients.

The overlapping number of potential candidate mRNAs and small RNAs identified among the seven patients was low (Figure 4). Thirty-four mRNA transcripts were found to overlap between pairs of patients. As an example, patients #1 and #2 showed an overlap with four genes (*TMSB4X, EEF1G, MGC16275, SNORD141B*). Patients #5, #6, and #7 (without tumor samples) showed an overlap of four genes (*SORL1, PTPRC, MIR6819, and NAMPT*), while #6 and #7 also shared five genes (*CELF2, EDEM3, SMCHD1, RAVER1, and CXCR1*).

Gene set enrichment analysis showed an up to 10-fold (*p* = 1.8 × 10^−6^) enrichment in the number of genes coding for biological and cellular processes associated with ribosomal RNA (rRNA) as well as a 7.5-fold significant (*p* = 7 × 10^−3^) enrichment in genes coding for T-cell activation (Table 3).

From the 231 identified genes (annotations are shown in Supplemental Table S2), 73 genes are already known to be related to GBM and biological processes, e.g., proliferation (n = 20), oncogenic biomarker (n = 13), immune and tumor microenvironment (n = 12), tumor suppression (n = 6), genes related to metastasis (n = 8), radio/chemoresistance genes (n = 9), stem cell niche genes (n = 5), and others (n = 158), Supplemental Table S2.

## 4. Discussion

GBM remains one of the most severe types of cancer. Using conventional imaging in order to differentiate treatment-related pseudoprogression and real tumor recurrence is challenging. The use of tumor-specific serological biomarkers, particularly mRNAs and miRNAs in peripheral blood, can provide a “fingerprint” at the gene expression level. mRNAs and miRNAs are crucial regulators that significantly influence the pathogenesis, development, and progression of cancer, as well as in the response to therapy [25]. Studies have shown that GBMs display distinct miRNA expression profiles related to proliferation, survival, invasion, angiogenesis, and stem cell-like characteristics [26]. The idea behind so-called “liquid biopsy” is that mRNAs and miRNAs can serve as surrogates for tumor masses in the body. Such an approach could complement (direct) tumor biopsy via surgery and MRI in monitoring GBM patients in terms of tumor recurrence and compensate for their inherent limitations. Early diagnosis and decision making for GBM recurrence might be more confident in clinical practice.

Most “liquid biopsy” studies focus on the isolation and analysis of exosomes out of plasma, which is laborious and time-consuming, resulting in low RNA yields [21,22,27] and requiring high blood volumes. In this prospective study, we used only 2.5 mL whole blood (PAXgene Blood RNA tubes, Qiagen, PreAnalytiX GmbH, Hilden, Germany) for detecting tumor-specific serological biomarkers as a more practical but probably less sensitive approach [10]. PAXgene Blood RNA Tubes include a unique reagent that stabilizes all RNA types and prevents ex vivo alterations in gene expression. This approach is intended to explore the possibility of omitting EV purification, making the interface to clinical applications more robust. However, “background noise” has to be expected when using whole blood because the whole blood might comprise tumor-specific biomarkers, among many others. Nevertheless, due to the complex isolation procedure and the low RNA yield from exosomes, pre-amplification is often necessary for subsequent gene expression analysis, despite the risk of amplification-induced bias [21,28,29].

Via whole-genome screening for each patient and applying a three-step filtering method to identify potential candidates for the identification of tumor recurrence, we found hundreds of candidate genes (mRNAs and miRNAs) for each patient. The filter criteria were chosen to minimize the noise inherent in using whole blood samples rather than isolating exosomes and to select potential candidate genes for each patient robustly. We selected genes expressed in both tumor tissues and blood samples pre-surgery to minimize the “noise” inherent in using whole blood samples. This filter criterion could be applied to four of the seven patients. In three patients, isolated RNA from tumor tissue was degraded and not suitable for further analysis. In this case, mRNAs/miRNAs in pre-surgery blood only were considered. Further, we assumed a downregulation (<2-fold) of candidates in the post-surgery phase and during the clinical course, reflecting the lack of tumor tissue. In case of tumor recurrence, we expected these gene expression measurements to revert to normal levels or even exceed them. In all seven patients, potential candidate genes could be identified, exhibiting the expected gene expression pattern during clinical follow-up. Patient #5 appeared as an “ideal” case in our “proof of concept” study, in which we were able to see an upregulation of tumor-specific serological biomarkers as an early indication of tumor recurrence preceding confirmation via MRI scan.

Interestingly, the decrease in DGE after excision of the tumor was emphasized at later time points after surgery. In concordance with a previous study, our results indicate that the acute effects of surgery are still evident in the early phase (5–7 days) after surgery and probably mask changes in gene expression [10]. This study confirmed that additional time points several weeks after surgery are necessary to identify suitable candidate genes.

Despite the large number of identified potential candidate genes for tumor recurrence diagnosis in each patient, the overlap of genes between the patients was low and indicated significant inter-individual variance among GBM patients [30]. This variability in the gene expression between different patients is a further hint to a personalized medical approach to optimize diagnosis and treatment. This prospective study supports the idea not only of individualized tumor therapy but also of individualized tumor diagnosis.

For mRNA analysis, we conducted an exon-based analysis for each patient and notably found that the overlap across different transcriptional candidate genes has been repeatedly identified on different exons within the same gene. For instance, several isoforms of *GLUL* (glutamine synthetase, n = 8), *SORL1* (sortilin-related receptor 1, n = 10), and *OAZ1* (ornithine decarboxylase antizyme 1, n = 5) could be identified (Supplemental Table S1). These redundancies in identifying the same potential candidate gene support our approach. Among the 231 identified genes, 73 were already identified to be related to GBM and tumor processes, such as proliferation, metastasis, tumor suppression, and others (for details, see Supplemental Table S2). However, most of the studies were conducted in cell lines ex vivo, which underscores the relevance of our in vivo examinations. Some genes appeared particularly interesting. For instance, Ferritin light chain (*FTL*) plays a crucial role in iron metabolism and has been linked to the survival of GBM patients. It was found that *FTL* expression was significantly increased in GBM patients compared to those with low-grade glioma [31,32]. In our study, *FTL* proved to be a promising candidate gene, showing deregulation in three patients (#1, #5, and #6). Additionally, *FTL* exhibited expression across various exons (up to four). Ferritin heavy chain 1 (*FTH1*), the iron regulatory protein, is increasingly linked to high tumor grade and poor survival outcomes in glioblastoma [32,33]. *FTH1* was also deregulated in three patients (#1, #4, and #6). The tumor suppressor gene *PTEN* (phosphate and tensin homolog) plays a crucial role by dephosphorylating *PIP3*. However, when *PTEN* is lost or downregulated, *PIP3* levels increase, triggering constant signaling and activation of the *PI3K/Akt* pathway. This promotes cell growth and is associated with a poor prognosis [34,35,36]. *PTEN* was deregulated in patient #5.

Certain candidate genes in our study, such as *let-7b* and *mir-125a*, were already recognized as biomarkers for GBM. *let-7b* and *mir-125a* (*mir-125a* was defined in low-risk compared to high-risk groups) were previously identified as highly expressed biomarkers in GBM patients [17]. Our findings indicated the deregulation of *let-7b* in two patients (#1 and #5), and *mir-125a* was deregulated in one patient, # 5. *miR-125a* has been identified as a tumor suppressor in GBM [37]. Furthermore, a significant decrease in the expression levels of *miR-454* in post-operative plasmas compared to pre-operative plasma was demonstrated. This miRNA is also a candidate in one patient (#1) in the current study [38].

*Mir-21* is described as an important miRNA investigated in cancer that has been found to be upregulated in both plasma and tissue of GBM patients, correlating with lower overall survival and tumor grading [39,40,41]. However, it was not detected in our cohort. There is almost no overlap between our potential candidate genes and the ones that are already mentioned in the literature.

In our prospective study, we faced challenges concerning the RIN values for some tissue material of the primary tumor. The isolated RNA was fully degraded in three patients. Despite the use of RNAlater solution, which is widely known for its ability to stabilize RNA and prevent degradation, these three tumor samples had to be excluded from further analysis. Also, the independent decision made by some patients to seek follow-up care at different institutions caused discrepancies between the total number of samples analyzed for each patient. Nevertheless, the seven patients included in our study provided an almost consistent set of samples. This forced us to apply different filter criteria for the selection of candidate RNAs.

Promising diagnostic biomarkers for tumor recurrence were identified. However, we can only speculate about the origin of the measured gene expression changes. Still, our novel triple-filter applied over the whole follow-up makes it likely that we selected the most meaningful genes out of thousands of candidate genes. The small number of patients examined is a limitation in our study. However, many genes with the potential to serve as a diagnostic marker in GBM patients with unclear tumor recurrence in the follow-up phase could be identified. This can be considered as a “proof of concept” and as an alternative to the laborious EV isolation liquid biopsy approach. Future work will require a larger sample size and using the proposed workflow.

Analysis of gene set enrichment consistently revealed significant involvements in rRNA in several functional categories. Rapid cell proliferation and a high rate of protein synthesis, which supports the growth and survival of the tumor, are associated with GBM [42]. The deregulation of ribosome biogenesis is associated with various cancers, including GBM, where ribosomal proteins promote tumor plasticity and stemness induction in glioma cells, emphasizing the critical role of rRNAs in tumorgenesis [43]. This was also demonstrated in our analysis.

Enrichment analysis identified the T-cell activation pathway among our differentially expressed genes. In CNS Grade 4 glioblastoma, the enrichment of T-cell activation pathways suggests these biomarkers may be involved in immune surveillance and response mechanisms within the GBM microenvironment, influencing tumor progression [44].

In summary, with our novel triple-filtering approach applied over the clinical follow-up, promising genes for the detection of tumor recurrence after initial therapy in patients with GBM WHO CNS Grade 4 could be identified, even before the confirmation of recurrence via MRI scan. Further research is required to independently validate the proposed workflow in whole blood as a robust alternative to the laborious isolation of exosomes. Our biomarker for tumor recurrence showed a significant inter-individual variance among GBM patients, thus supporting a personalized medical approach for optimized diagnosis findings. 

## 5. Conclusions

The utilization of liquid biopsy and circulating biomarkers holds significant promise as a minimally invasive approach for early diagnosis, prognosis, monitoring, and personalized therapy decisions in the context of tumor heterogeneity. Our prospective study highlighted the potential of tumor-derived serological biomarkers in the early detection of tumor recurrence in GBM. However, it is essential to note that further research is needed to understand the potential of these biomarkers and integrate them into clinical use.

## Figures and Tables

**Table 1:** Characteristics of the seven patients enrolled in this prospective study.

Patient ID	Sex	Age (Years)	Primary Tumor Tissue	Total No. of Blood Samples	Days of Blood Sampling		
					Pre-Surgery	Post-Surgery	Pre-/Post-Recurrence
1	F	65	yes	4	1	5, 84	3
2	M	37	yes	4	1	6, 28	1
3	M	60	yes	6	1	6, 19, 29, 97	0
4	M	79	yes	3	1	7	0
5	F	45	no	5	5	28, 97, 184	−13
6	M	60	no	5	1	9, 27	0, 14
7	M	59	no	6	1	7, 17, 23	4, 17

**Table 2:** Number of mRNAs and miRNAs in the study group.

**Patient ID**	**NGS**		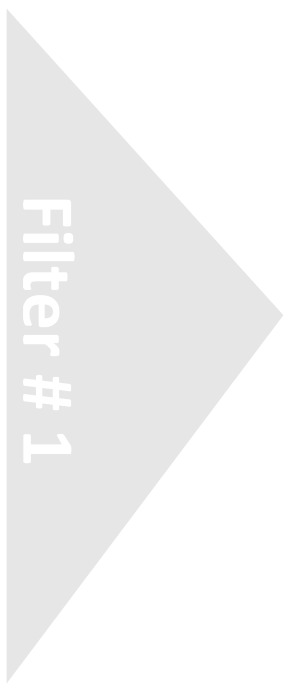	**Pre Surgery Phase**		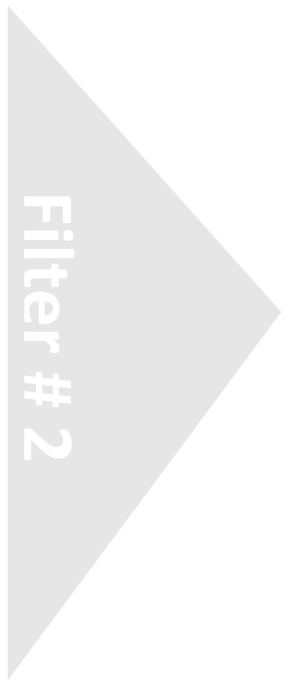	**Post Surgery Phase**		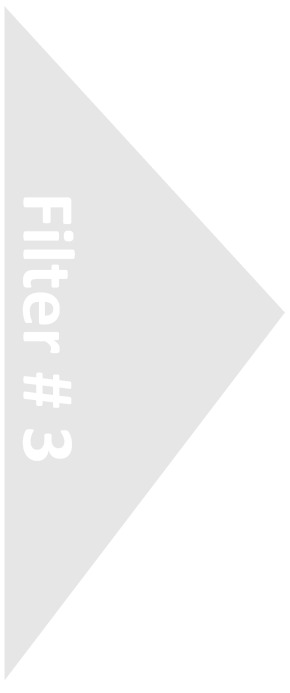	**Recurrence Phase**	
	**# mRNA**	**# miRNA**		**# mRNA**	**# miRNA**		**# mRNA**	**# miRNA**		**# mRNA**	**# miRNA**
1	01.4 × 10^5^	622		567	186		188	58		93	19
2	01.4 × 10^5^	622		500	187		81	41		27	1
3	01.4 × 10^5^	622		489	185		175	17		12	4
4	01.4 × 10^5^	622		492	196		135	43		6	10
5	01.4 × 10^5^	622		1194	243		504	68		72	15
6	01.4 × 10^5^	622		1117	242		370	46		88	9
7	01.4 × 10^5^	622		1240	236		566	42		76	14

**Table 3:** Gene set enrichment analysis. Genes are ordered with increasing *p*-values.

Classification	No. of Genes		Fold Enrichment	*p*-Value	FDR
	Observed	Expected			
**PANTHER GO-Slim Cellular Component**					
cytosolic ribosome	10	0.95	10.53	3.74 × 10^−8^	6.13 × 10^−6^
ribonucleoprotein complex	21	4.77	4.4	1.55 × 10^−8^	7.64 × 10^−6^
ribosome	12	1.5	8.02	3.59 × 10^−8^	8.84 × 10^−6^
ribosomal subunit	11	1.36	8.07	1.26 × 10^−7^	1.55 × 10^−5^
cytoplasm	91	58.09	1.57	1.77 × 10^−6^	1.75 × 10^−4^
cytosolic large ribosomal subunit	6	0.56	10.74	1.92 × 10^−5^	1.57 × 10^−3^
intracellular anatomical structure	124	93.38	1.33	4.68 × 10^−5^	3.29 × 10^−3^
intracellular organelle	101	73.07	1.38	1.11 × 10^−4^	6.84 × 10^−3^
large ribosomal subunit	6	0.8	7.46	1.54 × 10^−4^	8.40 × 10^−3^
cellular_component	160	132.78	1.2	2.72 × 10^−4^	1.12 × 10^−2^
small ribosomal subunit	5	0.56	8.95	2.34 × 10^−4^	1.15 × 10^−2^
Unclassified	70	97.22	0.72	2.72 × 10^−4^	1.22 × 10^−2^
organelle	101	75.66	1.33	5.16 × 10^−4^	1.95 × 10^−2^
cytosolic small ribosomal subunit	4	0.39	10.23	6.05 × 10^−4^	2.13 × 10^−2^
cellular anatomical entity	154	129.98	1.18	1.29 × 10^−3^	4.23 × 10^−2^
protein-containing complex	49	31.51	1.56	1.38 × 10^−3^	4.25 × 10^−2^
**PANTHER Protein Class**					
ribosomal protein	12	1.99	6.04	8.01 × 10^−7^	1.57 × 10^−4^
translational protein	14	3.74	3.74	2.62 × 10^−5^	2.57 × 10^−3^
**PANTHER Pathways**					
T cell activation	7	0.94	7.46	4.32 × 10^−5^	6.96 × 10^−3^
**GO molecular function complete**					
RNA binding	49	18.75	2.61	3.57 × 10^−10^	1.81 × 10^−6^
structural constituent of ribosome	15	1.88	7.99	7.07 × 10^−10^	1.79 × 10^−6^
binding	216	185.90	1.16	1.68 × 10^−8^	2.83 × 10^−5^
protein binding	196	161.51	1.21	1.34 × 10^−7^	1.70 × 10^−4^
Unclassified	6	25.60	0.23	2.43 × 10^−6^	2.47 × 10^−3^
molecular_function	224	204.40	1.10	2.43 × 10^−6^	2.06 × 10^−3^
organic cyclic compound binding	101	68.02	1.48	3.96 × 10^−6^	2.86 × 10^−3^
enzyme binding	46	23.61	1.95	9.00 × 10^−6^	5.71 × 10^−3^
structural molecule activity	24	8.89	2.70	1.05 × 10^−5^	5.93 × 10^−3^
nucleic acid binding	72	45.12	1.60	2.51 × 10^−5^	1.27 × 10^−2^
protein-containing complex binding	40	19.97	2.00	2.60 × 10^−5^	1.20 × 10^−2^
enzyme regulator activity	32	14.75	2.17	3.34 × 10^−5^	1.41 × 10^−2^
mRNA binding	14	3.84	3.64	3.50 × 10^−5^	1.37 × 10^−2^
immune receptor activity	9	1.70	5.30	5.44 × 10^−5^	1.97 × 10^−2^

## Data Availability

The data presented in this study are available on request from the corresponding author. The data are not publicly available due to privacy restrictions.

## Supplementary Materials

The following supporting information can be downloaded at: https://www.mdpi.com/article/10.3390/cancers16132345/s1, Table S1. Presents filtered mRNAs and miRNAs. miRNAs are presented as Unique Exact Mature 3′ and Unique Exact Mature 5′ (UEM3′ and UEM5′, respectively). A mature miRNA located closer (or equally close) to the 5′ end than to the 3′ end is referred to as “Mature 5′”; Table S2: Provides a summary of annotations for the identified genes, including gene names, symbols, persistent IDs, orthologs, Panther family/subfamily or Panther protein class, and their association with GBM. The analysis was conducted using PANTHER software.
