# Supplementary material for: Liquid Biopsy in Whole Blood for Identification of Gene Expression Patterns (mRNA and miRNA) Associated with Recurrence of Glioblastoma WHO CNS Grade 4
Source: Cancers (Basel). 2024 Jun 26;16(13):2345. doi: 10.3390/cancers16132345 (PMC11240769; doi:10.3390/cancers16132345)
Supplement: Supplementary file 1 [file cancers-16-02345-s001.zip › References for Supplemental Table S2.pdf]

References for Supplemental Table S2:

1. Zhang, X., et al., *Cathepsin B contributes to radioresistance by enhancing homologous recombination in glioblastoma.* Biomed Pharmacother, 2018. **107**: p. 390-396.
2. Ma, K., et al., *CTSB is a negative prognostic biomarker and therapeutic target associated with immune cells infiltration and immunosuppression in gliomas.* Sci Rep, 2022. **12**(1): p. 4295.
3. Gao, Q., et al., *Identifying Mutually Exclusive Gene Sets with Prognostic Value and Novel Potential Driver Genes in Patients with Glioblastoma.* Biomed Res Int, 2019. **2019**: p. 4860367.
4. Zhang, D., et al., *Inhibition of REST Suppresses Proliferation and Migration in Glioblastoma Cells.* Int J Mol Sci, 2016. **17**(5).
5. Kamal, M.M., et al., *REST regulates oncogenic properties of glioblastoma stem cells.* Stem Cells, 2012. **30**(3): p. 405-14.
6. Zhang, R., et al., *MicroRNA-140-5p suppresses invasion and proliferation of glioma cells by targeting glutamate-ammonia ligase (GLUL).* Neoplasma, 2020. **67**(2).
7. Cheng, Y.W., et al., *miR-3059-3p Regulates Glioblastoma Multiforme Radiosensitivity Enhancement through the Homologous Recombination Pathway of DNA Repair.* J Oncol, 2022. **2022**: p. 7250278.
8. Hu, S., et al., *RPS14 promotes the development and progression of glioma via p53 signaling pathway.* Experimental Cell Research, 2023. **423**(1): p. 113451.
9. Vidomanova, E., et al., *The effect of temozolomide on apoptosis-related gene expression changes in glioblastoma cells.* Bratisl Lek Listy, 2022. **123**(4): p. 236-243.
10. Wu, T., et al., *Expression of Ferritin Light Chain (FTL) Is Elevated in Glioblastoma, and FTL Silencing Inhibits Glioblastoma Cell Proliferation via the GADD45/JNK Pathway.* PLoS One, 2016. **11**(2): p. e0149361.
11. Yang, B., et al., *Proteomic screening and identification of microRNA-128 targets in glioma cells.* Proteomics, 2015. **15**(15): p. 2602-17.
12. Fei, X., et al., *TRIM22 orchestrates the proliferation of GBMs and the benefits of TMZ by coordinating the modification and degradation of RIG-I.* Mol Ther Oncolytics, 2022. **26**: p. 413-428.
13. Guo, Q., et al., *NAMPT: A potential prognostic and therapeutic biomarker in patients with glioblastoma.* Oncol Rep, 2019. **42**(3): p. 963-972.
14. Gont, A., et al., *PREX1 integrates G protein-coupled receptor and phosphoinositide 3-kinase signaling to promote glioblastoma invasion.* Oncotarget, 2017. **8**(5): p. 8559-8573.
15. Sun, S., et al., *GPBAR1 promotes proliferation and is related to poor prognosis of high-grade glioma via inducing MAFB expression.* Histol Histopathol, 2022. **37**(3): p. 251-260.
16. Sharma, N., et al., *Differential Expression Profile of NLRs and AIM2 in Glioma and Implications for NLRP12 in Glioblastoma.* Sci Rep, 2019. **9**(1): p. 8480.
17. Yin, X., et al., *Identification of novel prognostic targets in glioblastoma using bioinformatics analysis.* Biomed Eng Online, 2022. **21**(1): p. 26.
18. Zhu, C., et al., *IFI30 Is a Novel Immune-Related Target with Predicting Value of Prognosis and Treatment Response in Glioblastoma.* Onco Targets Ther, 2020. **13**: p. 1129-1143.
19. Moretti, I.F., et al., *Late p65 nuclear translocation in glioblastoma cells indicates non-canonical TLR4 signaling and activation of DNA repair genes.* Sci Rep, 2021. **11**(1): p. 1333.
20. Tassinari, V., et al., *ADAR1 is a new target of METTL3 and plays a pro-oncogenic role in glioblastoma by an editing-independent mechanism.* Genome Biol, 2021. **22**(1): p. 51.
21. Yao, Y., et al., *Down-regulation of ribosomal protein S15A inhibits proliferation of human glioblastoma cells in vivo and in vitro via AKT pathway.* Tumour Biol, 2016. **37**(4): p. 4979-90.
22. Jie, C., et al., *AdipoR2 inhibits human glioblastoma cell growth through the AMPK/mTOR pathway.* Eur J Med Res, 2021. **26**(1): p. 28.
23. Han, X., et al., *Mechanism of microRNA-431-5p-EPB41L1 interaction in glioblastoma multiforme cells.* Arch Med Sci, 2019. **15**(6): p. 1555-1564.

24. Gao, Y.F., et al., *LncRNA FOXD1-AS1 acts as a potential oncogenic biomarker in glioma.* CNS Neurosci Ther, 2020. **26**(1): p. 66-75.
25. Cheng, P., et al., *FOXD1-ALDH1A3 Signaling Is a Determinant for the Self-Renewal and Tumorigenicity of Mesenchymal Glioma Stem Cells.* Cancer Res, 2016. **76**(24): p. 7219-7230.
26. Huntsman, D.G., et al., *MLL2, the second human homolog of the Drosophila trithorax gene, maps to 19q13.1 and is amplified in solid tumor cell lines.* Oncogene, 1999. **18**(56): p. 7975-84.
27. Su, R., et al., *PABPC1-induced stabilization of BDNF-AS inhibits malignant progression of glioblastoma cells through STAU1-mediated decay.* Cell Death Dis, 2020. **11**(2): p. 81.
28. Anvari, K., et al., *P04.20 Analysis of HORMAD1, FTHL17 and ADAM29 Cancer/testis specific genes expression in Glioblastoma*. Neuro Oncol. 2018 Sep;20(Suppl 3):iii282-3. doi: 10.1093/neuonc/noy139.254. Epub 2018 Sep 19.
29. Xu, H., et al., *Molecular and clinical characterization of PARP9 in gliomas: A potential immunotherapeutic target.* CNS Neurosci Ther, 2020. **26**(8): p. 804-814.
30. Okura, H., et al., *A role for activated Cdc42 in glioblastoma multiforme invasion.* Oncotarget, 2016. **7**(35): p. 56958-56975.
31. Baeza-Kallee, N., et al., *Deciphering the Action of Neuraminidase in Glioblastoma Models.* Int J Mol Sci, 2023. **24**(14).
32. Cheng, W.-Y., et al., *Luteolin inhibits migration of human glioblastoma U-87 MG and T98G cells through downregulation of Cdc42 expression and PI3K/AKT activity.* Molecular Biology Reports, 2013. **40**(9): p. 5315-5326.
33. Xu, H., et al., *High expression of WDR1 in primary glioblastoma is associated with poor prognosis.* Am J Transl Res, 2016. **8**(2): p. 1253-64.
34. Fan, Y., et al., *Profilin-1 phosphorylation directs angiocrine expression and glioblastoma progression through HIF-1α accumulation.* Nat Cell Biol, 2014. **16**(5): p. 445-56.
35. Syafruddin, S.E., et al., *Integration of RNA-Seq and proteomics data identifies glioblastoma multiforme surfaceome signature.* BMC Cancer, 2021. **21**(1): p. 850.
36. Guo, X., et al., *Overexpression of microRNA-129-5p in glioblastoma inhibits cell proliferation, migration, and colony-forming ability by targeting ZFP36L1.* Bosn J Basic Med Sci, 2020. **20**(4): p. 459-470.
37. Knobbe, C.B., J. Reifenberger, and G. Reifenberger, *Mutation analysis of the Ras pathway genes NRAS, HRAS, KRAS and BRAF in glioblastomas.* Acta Neuropathol, 2004. **108**(6): p. 467-70.
38. MacDonald, T.J., et al., *Progression-associated genes in astrocytoma identified by novel microarray gene expression data reanalysis.* Methods Mol Biol, 2007. **377**: p. 203-22.
39. Jiang, J., et al., *Influence of SLC40A1 on Cytokine Interactions and Immune Infiltration in Glioblastoma.* Neuromolecular Med, 2024. **26**(1): p. 21.
40. Wu, Y., et al., *EDEM2 is a diagnostic and prognostic biomarker and associated with immune infiltration in glioma: A comprehensive analysis.* Front Oncol, 2022. **12**: p. 1054012.
41. Peng, Q., et al., *Prediction of a competing endogenous RNA co-expression network as a prognostic marker in glioblastoma.* J Cell Mol Med, 2020. **24**(22): p. 13346-13355.
42. Xu, H., et al., *Prognostic and predictive value of FCER1G in glioma outcomes and response to immunotherapy.* Cancer Cell Int, 2021. **21**(1): p. 103.
43. Xu, S., et al., *CD74 Correlated With Malignancies and Immune Microenvironment in Gliomas.* Front Mol Biosci, 2021. **8**: p. 706949.
44. Hueng, D.Y., et al., *DDX3X Biomarker Correlates with Poor Survival in Human Gliomas.* Int J Mol Sci, 2015. **16**(7): p. 15578-91.
45. Ren, W., W. Jin, and Z. Liang, *Construction and Validation of an Immune-Related Risk Score Model for Survival Prediction in Glioblastoma.* Front Neurol, 2022. **13**: p. 832944.
46. Liang, Y., A.W. Bollen, and N. Gupta, *CC chemokine receptor-2A is frequently overexpressed in glioblastoma.* J Neurooncol, 2008. **86**(2): p. 153-63.
47. Haghparast, A., M. Heidari Kharaji, and A.M. Malvandi, *Down-regulation of CD14 transcripts in human glioblastoma cell line U87 MG.* Iran J Immunol, 2011. **8**(2): p. 111-9.

48. Malvandi, A.M., J. Mehrzad, and M.S. Moghaddam, *Gene Expression Quantification of Toll like Receptors 2, 4 and Co-molecules in Human Glioblastoma Cell Line (U87-MG): Toward a New In vitro Model of Inflammation.* Iran J Basic Med Sci, 2011. **14**(5): p. 428-35.
49. Li, J., et al., *Integrative analysis of metabolism subtypes and identification of prognostic metabolism-related genes for glioblastoma.* Biosci Rep, 2024. **44**(3).
50. Wang, L., et al., *Screening of differentially expressed genes associated with human glioblastoma and functional analysis using a DNA microarray.* Mol Med Rep, 2015. **12**(2): p. 1991-6.
51. Oji, Y., et al., *The translation elongation factor eEF2 is a novel tumor-associated antigen overexpressed in various types of cancers.* Int J Oncol, 2014. **44**(5): p. 1461-9.
52. Fossdal, G., et al., *Aqp 9 and brain tumour stem cells.* ScientificWorldJournal, 2012. **2012**: p. 915176.
53. Pal, J., et al., *Epigenetically silenced GNG4 inhibits SDF1α/CXCR4 signaling in mesenchymal glioblastoma.* Genes Cancer, 2016. **7**(3-4): p. 136-47.
54. Giotta Lucifero, A. and S. Luzzi, *Immune Landscape in PTEN-Related Glioma Microenvironment: A Bioinformatic Analysis.* Brain Sci, 2022. **12**(4).
55. Wang, X.P., X.L. Deng, and L.Y. Li, *MicroRNA-584 functions as a tumor suppressor and targets PTTG1IP in glioma.* Int J Clin Exp Pathol, 2014. **7**(12): p. 8573-82.
56. Oldrini, B., et al., *EGFR feedback-inhibition by Ran-binding protein 6 is disrupted in cancer.* Nat Commun, 2017. **8**(1): p. 2035.
57. Que, Z., et al., *Dihydroartemisinin inhibits EMT of glioma via gene BASP1 in extrachromosomal DNA.* Biochem Biophys Res Commun, 2023. **675**: p. 130-138.
58. Zhang, P., et al., *A novel indication of thioredoxin-interacting protein as a tumor suppressor gene in malignant glioma.* Oncol Lett, 2017. **14**(2): p. 2053-2058.
59. Jiang, L., et al., *Bioinformatics Analysis Discovers Microtubular Tubulin Beta 6 Class V (TUBB6) as a Potential Therapeutic Target in Glioblastoma.* Front Genet, 2020. **11**: p. 566579.
60. Han, T., et al., *Comprehensive Analysis of Inflammatory Response–Related Genes, and Prognosis and Immune Infiltration in Patients With Low-Grade Glioma.* Frontiers in Pharmacology, 2021. **12**.
61. Aroca, F., et al., *Expression of PECAM-1/CD31 isoforms in human brain gliomas.* J Neurooncol, 1999. **43**(1): p. 19-25.
62. Wang, T., et al., *LINC01116 promotes tumor proliferation and neutrophil recruitment via DDX5-mediated regulation of IL-1β in glioma cell.* Cell Death & Disease, 2020. **11**(5): p. 302.
63. Gupta, M.K., et al., *Altered transcriptional regulatory proteins in glioblastoma and YBX1 as a potential regulator of tumor invasion.* Sci Rep, 2019. **9**(1): p. 10986.
64. Sharma, I., et al., *IL-8/CXCR1/2 signalling promotes tumor cell proliferation, invasion and vascular mimicry in glioblastoma.* J Biomed Sci, 2018. **25**(1): p. 62.
65. Wen, X., et al., *Identification of key genes and their microRNAs in glioma: evidence from bioinformatic analysis.* 2020.
66. Sun, Z., et al., *Forkhead box P3 regulates ARHGAP15 expression and affects migration of glioma cells through the Rac1 signaling pathway.* Cancer Sci, 2017. **108**(1): p. 61-72.
67. Li, S., et al., *FBXO7 Confers Mesenchymal Properties and Chemoresistance in Glioblastoma by Controlling Rbfox2-Mediated Alternative Splicing.* Adv Sci (Weinh), 2023. **10**(33): p. e2303561.
68. Liu, Y., et al., *Bioinformatics analysis of microenvironment-related genes associated with radioresistance in glioblastoma.* Transl Cancer Res, 2020. **9**(12): p. 7495-7504.
69. Kang, W., et al., *Talin1 targeting potentiates anti-angiogenic therapy by attenuating invasion and stem-like features of glioblastoma multiforme.* Oncotarget, 2015. **6**(29): p. 27239-51.
70. Lin, W., et al., *VMP1, a novel prognostic biomarker, contributes to glioma development by regulating autophagy.* J Neuroinflammation, 2021. **18**(1): p. 165.
71. Cho, Y.J., et al., *Involvement of DDX6 gene in radio- and chemoresistance in glioblastoma.* Int J Oncol, 2016. **48**(3): p. 1053-62.

72. Arya, K.R., et al., *Identification of Hub Genes and Key Pathways Associated with Anti-VEGF Resistant Glioblastoma Using Gene Expression Data Analysis.* Biomolecules, 2021. **11**(3).
73. Fuentes-Fayos, A.C., et al., *SF3B1 inhibition disrupts malignancy and prolongs survival in glioblastoma patients through BCL2L1 splicing and mTOR/ß-catenin pathways imbalances.* J Exp Clin Cancer Res, 2022. **41**(1): p. 39.
74. Yi, G.Z., et al., *Identification of Key Candidate Proteins and Pathways Associated with Temozolomide Resistance in Glioblastoma Based on Subcellular Proteomics and Bioinformatical Analysis.* Biomed Res Int, 2018. **2018**: p. 5238760.
75. Chien, C.H., et al., *Enrichment of superoxide dismutase 2 in glioblastoma confers to acquisition of temozolomide resistance that is associated with tumor-initiating cell subsets.* J Biomed Sci, 2019. **26**(1): p. 77.
76. Sun, C., et al., *Alteration of DNA damage signaling pathway profile in radiation-treated glioblastoma stem-like cells.* Oncol Lett, 2015. **10**(3): p. 1769-1774.
77. Turchi, L., et al., *CELF2 Sustains a Proliferating/OLIG2+ Glioblastoma Cell Phenotype via the Epigenetic Repression of SOX3.* Cancers (Basel), 2023. **15**(20).
78. Ravi, V., et al., *Liposomal delivery of ferritin heavy chain 1 (FTH1) siRNA in patient xenograft derived glioblastoma initiating cells suggests different sensitivities to radiation and distinct survival mechanisms.* PLoS One, 2019. **14**(9): p. e0221952.
79. Liu, J., et al., *ZNF117 regulates glioblastoma stem cell differentiation towards oligodendroglial lineage.* Nat Commun, 2022. **13**(1): p. 2196.
80. Park, S., et al., *Suppression of Glioblastoma Stem Cell Potency and Tumor Growth via LRRK2 Inhibition.* Int J Stem Cells, 2024.
